# Evaluating the Clinical Risk Factors Associated With Miscarriages in Women in Karachi, Pakistan

**DOI:** 10.7759/cureus.19057

**Published:** 2021-10-26

**Authors:** Zafar Iqbal, Syed Daniyal A Jilanee, Lalitha P Uppada, Samahir Imtiaz, Hamadullah Khan, Syed Muhammad Huzaifa Shah, Sohaib Tousif, Anum Rahim

**Affiliations:** 1 Emergency Medicine, California Institute of Behavioral Neurosciences and Psychology, Fairfield, USA; 2 Emergency Department, The Kidney Center, Karachi, PAK; 3 Medical College, Liaquat National Hospital and Medical College, Karachi, PAK; 4 Medical College, Gandhi Medical College, Hyderabad, IND; 5 Medical School, Ziauddin University, Karachi, PAK; 6 Medical Education, Ziauddin University, Karachi, PAK; 7 Medical School, Ziauddin Medical University, Karachi, PAK; 8 Indus Hospital Research Center, The Indus Hospital, Karachi, PAK

**Keywords:** risk analysis, pregnancy loss, pakistan, risk factors, miscarriages

## Abstract

Introduction

Spontaneous pregnancy loss is unquestionably common worldwide, with roughly 5% of pregnancies ending in this way. Miscarriage can lead to serious psychological issues for women as well as their mothers. Although, it is irreversible but can be prevented through proper risk assessment of women. The goal of this study is to find clinical predictors of miscarriages in Karachi, Pakistani women.

Methodology

The study is a retrospective chart review that used data of women having livebirth and miscarriages at the Liaquat National Hospital Karachi Pakistan. Data of a total of 517 women were included in the study, out of which 453 have had a live birth, and 64 had miscarriages. To determine the factors associated with miscarriages, multivariable logistic regression was used.

Results

The mean age of women was 31.08 (±5.10) years. Age of mother over 40 years (adjusted odds ratio [AOR]=10.28; p-value=0.001), overweight and obesity (AOR=3.01; p-value=0.001) and history of miscarriage (AOR=2.91; p-value=0.003) are variables significantly associated with miscarriages.

Conclusion

Findings of the current study shown that risk factors of miscarriages included age of mother, increased BMI and previous history of miscarriages. All these factors need to be considered while providing antenatal care to mothers to mitigate the risk of miscarriages.

## Introduction

Spontaneous pregnancy loss is undoubtedly common worldwide, where nearly 5% of pregnancies end by spontaneous loss [1]. Miscarriage is considered irreversible, and prevention is only a possible means to intervene in this that has serious psychological outcomes for women as well as for their family members, and it can also lead to delay in successful childbearing [2]. Spontaneous abortion has a multifactorial origin of both non-genetic and genetic causes that can be interlinked. Polymorphisms and chromosomal abnormalities stand out among the genetic factors, while as non-genetic factors, thrombophilic disorders, life history, endocrine disorders, occupational causes, socioeconomic status, environmental causes, and infectious agents stand out [3,4]. It is predicted that one-quarter of spontaneous abortions can be prevented if risk factors can be mitigated [5].

Past studies have explored the risk factors of miscarriages, including infertility, polycystic ovarian syndrome (PCOS), smoking, body mass index (BMI), and age in 1,196 pregnant women. The study found that the rate of pregnancy loss was 16% before the sixth to seventh week of pregnancy. In addition, smoking is associated with an enhanced risk of early pregnancy loss. On the other hand, obesity and age did not significantly correlate with the increased risk of early pregnancy loss [6]. One of the factors that enhance the risk of miscarriages is the previous miscarriage history. The fact that females are increasingly delaying pregnancy into their 30s exacerbates this clinical situation. As a result, even one miscarriage can impair a couple's ability to reproduce successfully in the future [7].

Individual risk factors for miscarriage have been thoroughly investigated, but prevention of miscarriages remains mainly unexplored; doctors mostly lack access to a validated collection of risk factors, particularly in pregnant women who have a low risk of loss early in pregnancy [8]. Besides this, clinical factors associated with early pregnancy need to be explored in developing countries. Certain factors are not in control, but understanding the significance of these factors in spontaneous pregnancy loss and identification of women at high risk of pregnancy loss, effective counseling, and antenatal care services can be promoted in order to prevent pregnancy loss among high-risk women. We can predict the likelihood of miscarriage based on individual parameters and laboratory test results in order to carry out preventive and targeted interventions to prevent miscarriage and to inform patients in advance of the risk of miscarriage in order to avoid patients having a low awareness of unpredictable pregnancy risks, resulting in anxiety and distrust between patients and doctors. The aim of this study is to determine the clinical predictors linked to miscarriages in women in Karachi, Pakistan.

## Materials and methods

It was a retrospective chart review including data of women with livebirth and miscarriage presented at the Liaquat National Hospital Karachi, Pakistan, from 1 January 2019 to 31 December 2020. Liaquat National Hospital is a teaching hospital in Karachi with a total capacity of nearly 500 beds. It includes major specialties such as obstetrics and gynecology, pediatrics, internal medicine, general surgery, and orthopedics. 

The data for this study were retrieved from Hospital Management Information System (HMIS) by the co-investigator. All data were extracted into the Microsoft Excel file and reviewed for any missing values. Patients with incomplete data were not included in the final analysis. Women with any kind of chromosomal or genetic disorder were excluded from the study, while women with stillbirths were also not included in the final analysis. Data of a total of 517 women were included in the study, out of which 453 have had a live birth, and 64 had miscarriages. Variables extracted from HMIS included age of women at the time of pregnancy, parity (categorized into three groups including 0, 1 and 2 or more, BMI, history of miscarriage, Gestational diabetes mellitus (GDM), diabetes mellitus (DM), hypertension, history of C-section delivery and history of premature delivery. The primary outcome variable was the pregnancy outcome categorized into two groups including "Miscarriage" and "live birth".

Statistical analysis

All collected data were analyzed using STATA Version 16.0 (College Station, TX: StataCorp LLC). Descriptive statistics were presented as the mean and standard deviation for continuous variables, while percentages and frequencies were calculated for categorical variables. To assess the relationship of outcome with independent continuous and categorical variables, an independent t-test and Chi-square test were used as univariate analysis. p-value <0.25 was considered as the standard level of significance. Variables significant in univariate analysis were used to build a final model using multivariable logistic regression to adjust confounding variables. At multivariable analysis, a cut-off of p-value was kept at 0.05. 

## Results

Overall, 517 women were included in the final analysis. Table 1 shows the characteristics of study participants. The mean age of women was 31.08 (±5.10) years. The majority of women had a normal BMI (52.03%), while 44.87% of women were either overweight or obese. In relation to comorbidities, 18.76% of women had hypertension, 7.56% had diabetes, and 9.86% had hypothyroidism. 12.21 of women had a history of miscarriages.

**Table 1:** Characteristics of study participants. *Mean (SD).

Variable	n (%)
Age*	31.08 (5.10)
Parity	
0	151 (29.21)
1	167 (32.30)
2 or more	199 (38.49)
BMI	
Underweight	16 (3.09)
Normal	269 (52.03)
Overweight/obese	232 (44.87)
Diabetes	
Yes	39 (7.56)
No	477 (92.44)
History of Miscarriage	
No	453 (87.79)
Yes	63 (12.21)
History of C-section	
Yes	161 (31.20)
No	355 (68.80)
Hypertension	
Yes	97 (18.76)
No	420 (81.24)
Hypothyroidism	
Yes	51 (9.86)
No	466 (90.14)

To determine the factors associated with miscarriages among women in Karachi, Pakistan, firstly a univariate analysis was run. Table 2 shows the result of the univariate analysis. Factors associated with miscarriages including age of mother (p-value=0.001), history of miscarriage (p-value=0.001), BMI of mother (p-value=0.001), hypertension (p-value= 0.088) and history of premature deliver (p-value=0.214).

**Table 2:** Comparison of participants characteristics with pregnancy outcomes. *Significant at p-value<0.25.

Variable	Livebirth	Miscarriages	p-value
Age			
less than 30 years	194 (42.83)	18 (28.13)	0.001*
30-35 years	158 (34.88)	25 (39.06)	
35-40 years	89 (19.65)	9 (14.06)	
More than 40 years	12 (2.65)	12 (18.75)	
Parity			
0	136 (30.02)	15 (23.44)	0.313
1	148 (32.67)	19 (29.69)	
2 or more	169 (37.31)	30 (46.88)	
Hypothyroidism			
Yes	45 (9.93)	6 (9.38)	0.888
No	408 (90.07)	58 (90.63)	
Diabetes			
Yes	34 (7.52)	5 (7.81)	0.934
No	418 (92.48)	59 (92.19)	
History of miscarriage			
No	405 (89.60)	48 (75.00)	0.001*
Yes	47 (10.40)	16 (25.00)	
History of premature delivery			
No	419 (92.70)	62 (96.88)	0.214*
Yes	33 (7.30)	2 (3.13)	
Hypertension			
Yes	80 (7.66)	17 (26.56)	0.088
No	373 (82.34)	47 (73.44)	
BMI			
Underweight	15 (3.31)	1 (1.56)	0.001*
Normal	250 (55.19)	19 (29.69)	
Overweight/obese	188 (41.50)	44 (68.75)	
History of C-section			
Yes	145 (32.08)	16 (25.00)	0.253
No	307 (67.92)	48 (75.00)	

These significant variables in the univariate analysis were used in the multivariable logistic regression to determine their relationship with miscarriages after controlling with confounding variables. In the end, the final model was developed that consisted of one outcome variable and three independent significant risk factors affecting the outcome, as shown in Table 3.

**Table 3:** Multivariable logistic regression of factors associated with miscarriages. AOR: adjusted odds ratio.

Variable	AOR (95% CI)	p-value
Age		
less than 30 years		
30-35 years	1.54 (0.79-2.98)	0.201
35-40 years	0.92 (0.38-2.17)	0.851
More than 40 years	10.28 (3.83-27.61)	0.001
History of miscarriage		
No		
Yes	2.91 (1.44-5.87)	0.003
BMI		
Underweight	1.25 (0.15-10.20)	0.833
Normal		
Overweight/obese	3.18 (1.75-5.79)	0.001

Firstly, age is found to be a significant variable. The odds of miscarriage are 10.28 times in mothers with the age of 40 years or older as compared to younger mothers (AOR=10.28; p-value=0.001) after adjusting all other independent variables. Secondly, women who are overweight or obese are more likely to have a miscarriage than mothers with normal BMI (AOR=3.01; p-value=0.001). Lastly, the history of miscarriage is another variable found to be significantly associated with the outcome. It means that mothers with a previous history of miscarriage are more likely to have a miscarriage (AOR=2.91; p-value=0.003).

## Discussion

Miscarriage is defined as the natural death of a fetus before its ability to survive independently [9]. There are certain factors that increase the risk of miscarriages, including drug addiction, obesity, smoking, history of miscarriages, diabetes, and increased age of mother [10]. Loss of pregnancy is about 45% in women over the age of 40 years and about 10% in women under the age of 35 years [9]. The study conducted by Hur et al. found that thin endometrium, elevated levels of FSH, elevated levels of basal estradiol, and age of mother are significantly associated with the early loss of pregnancy among women undergoing assisted reproductive technology (ART) [11]. The current study was conducted with the aim to identify factors associated with miscarriages among women in Karachi, Pakistan. Factors that were significantly associated with miscarriages in the current study include the age of the mother over 40 years, obesity, and previous history of miscarriages.

Given the global trend of delaying childbearing, it is vital to investigate the impact of age on women's reproductive potential, even if it is not yet fully understood. It is widely known that after the age of 20 years, a woman's fertility begins to diminish gradually and then rapidly around the age of 35 years [12]. A reduction in oocyte quality and less receptive endometrium can be accountable for the enhanced miscarriage frequency in fading reproductive years. Increased age of mothers can also account for inadequate progesterone concentration, and it has been proposed that the corpus luteum and fertilized oocytes produce inadequate progesterone in order to endure implantation at the time of senescence [13].

Our study has found that women with higher BMI are at greater risk of miscarriages. The findings of the current study are consistent with the study conducted by Arck et al. [13]. Over the past few years, both underweight and overweight have been studied extensively in relation to their reproductive ability [14]. The study conducted by Arck et al. found that Women who were quite slim, with a BMI of less than 20 kg/m^2^, had a higher risk of miscarriage, particularly during the first seven weeks of pregnancy, regardless of their age. The relationship between BMI and blood leptin levels is well-known [13]. Adipose tissue secretes leptin hormone, and it has an important role in body weight regulation through the development of a feedback loop between the hypothalamic centers and the energy reserves that control the food intake. As a result, it is believed that leptin serves as a permissive factor, allowing the initiation of energy-demanding events like pregnancy only when the energy reserve is sufficient to ensure its success [15].

Previous studies have shown that multiple pregnancies can enhance the risk of miscarriages that is linked to a rise in the fetuses' number [16]. However, our study did not show any significant association between parity and miscarriages. Besides this, the study conducted by Mills et al. showed that the risk of miscarriages increased among women with diabetes mellitus [17], while no significant association was found between miscarriages and diabetes mellitus in the current study. Another important factor significantly associated with miscarriages included a past history of miscarriages that is consistent with the findings of the study conducted by Zargar et al. [18]. The study found that women with a previous history of miscarriages and abortion are at greater risk of miscarriages. Therefore, proper screening of these women is considered important in the clinical setting to reduce or eliminate the risk of unsuccessful pregnancy outcomes. Even though smoking is an important risk factor of miscarriages, but in our study, all women were non-smokers. Hence, the relationship cannot be determined. However, in past studies, a significant association was found between smoking and the risk of miscarriages [16]. Studies have also found a link between smoking and miscarriage. Tobacco smoke contains carbon monoxide, which can prevent a developing fetus from getting adequate oxygen. Tobacco smoke carries additional toxins that are harmful to unborn children [19].

The study has certain limitations. Firstly, the data were collected retrospectively, so most of the important variables such as socioeconomic status, education were not included in the analysis. Secondly, the study included the data of only one hospital. In the future, more studies need to be conducted in the region, including follow-up studies to identify all possible risk factors of miscarriages among women in Pakistan in order to design effective therapeutic interventions to reduce the incidence of miscarriages. The current study provides important information to health care professionals by redefining groups of apparently low-risk females in relation to the increased but ignored miscarriage risk. Considering the findings of the current study as well as the existing data from basic epidemiology and science, it is important to note that miscarriage occurs not because of a single factor, but it happens due to interaction of different psychological, physiological, anamnestic, and demographic factors.

## Conclusions

The current study has shown that the increased age of the female, increased BMI and past history of miscarriages are significant factors affecting the risk of miscarriages. All these factors can be detected at regular doctor visits. Clinicians should conduct thorough physical, psychological and demographic factors assessment to identify high-risk women at an earlier stage in order to plan interventions and treatment to mitigate that risk. These insights can help health care professionals to recognize pregnant women who need additional therapeutic interventions and monitoring like progesterone supplementation at the time of early pregnancy.
